# PFKFB3-Mediated Glycolytic Metabolic Reprogramming Regulates Inflammatory Response in Dry Eye Disease

**DOI:** 10.1167/iovs.66.11.76

**Published:** 2025-08-29

**Authors:** Kaiye Zhang, Yu Zhang, Xiaojie Wan, Yujie Mou, Xiaodan Huang

**Affiliations:** 1 Eye Center, The Second Affiliated Hospital, School of Medicine, Zhejiang University, Zhejiang Provincial Key Laboratory of Ophthalmology, Zhejiang Provincial Clinical Research Center for Eye Diseases, Zhejiang Provincial Engineering Institute on Eye Diseases, Hangzhou, China; 2 Department of Ophthalmology, Beijing Key Laboratory of Restoration of Damaged Ocular Nerve, Peking University Third Hospital, Beijing, China

**Keywords:** dry eye disease, glycolysis, PFKFB3, NF-κB signaling pathway

## Abstract

**Purpose:**

To investigate glycolytic and inflammatory changes on the ocular surface caused by dry eye disease (DED) and the regulatory effect of 6-phosphofructo-2-kinase/fructose-2,6-biphosphatase 3 (PFKFB3)-dependent glycolysis on the nuclear factor kappa B (NF-κB) pathway.

**Methods:**

Reverse transcription quantitative polymerase chain reaction (RT-qPCR) and a lactate assay were used to evaluate the expression of glycolytic genes, lactate secretion, and inflammatory factors in human corneal epithelial cells (HCECs) under hyperosmotic conditions, which served as an in vitro DED model. Transcriptome sequencing identified key regulatory genes in HCECs under hyperosmotic stimulation. *PFKFB3* overexpression plasmids and the small molecule inhibitor 3-(3-pyridinyl)-1-(4-pyridinyl)-2-propen-1-one or small interfering RNA (siRNA) were used to validate the role of PFKFB3 in glycolytic reprogramming and NF-κB pathway activation.

**Results:**

Hyperosmotic stress significantly upregulated glycolytic metabolic enzymes, increased lactate production, and induced inflammatory cytokine secretion in HCECs. Transcriptomics revealed a marked upregulation of the glycolytic regulator *PFKFB3* and NF-κB–related genes. Overexpression of *PFKFB3* further enhanced NF-κB pathway activation. Inhibition of *PFKFB3* reversed hyperosmotic-induced glycolytic activation, suppressed NF-κB phosphorylation, and reduced tumor necrosis factor alpha (TNF-α) secretion.

**Conclusions:**

Hyperosmotic stress activated the NF-κB pathway through PFKFB3-dependent glycolytic reprogramming, forming a vicious metabolic-inflammatory cycle. Targeting PFKFB3 may block this interaction and provide a novel therapeutic strategy for DED.

Dry eye disease (DED), a highly prevalent ocular surface disorder, has a global prevalence rate ranging from 5% to 50%.^1^ It is characterized by symptoms such as dryness, redness, pain, and blurred vision, and, in severe cases, it can lead to corneal ulcers and scarring.^2^^,^^3^ With the widespread use of smartphones, this condition poses an even greater public health burden.^4^^,^^5^^,^^6^ Traditional pharmacological treatments, including artificial tears, steroids, and immunosuppressants, only alleviate symptoms, and some may cause adverse effects with long-term use.^7^ Consequently, there is an urgent need to explore the mechanisms of DED to identify more effective therapies. In 2017, the International Tear Film and Ocular Surface Association published the International Dry Eye Workshop Report, which defined DED as a multifactorial disease of the ocular surface characterized primarily by an imbalance in tear film homeostasis. The core mechanisms underlying DED include tear film hyperosmolarity, tear film instability, ocular surface inflammation and damage, and neurosensory abnormalities.^8^ Hyperosmolarity triggers epithelial cell apoptosis and the release of inflammatory factors through pathways such as the mitogen-activated protein kinase (MAPK) pathway,^9^^–^^15^ forming a vicious cycle of “hyperosmolarity–inflammation–ocular surface damage.”^16^ However, the specific mechanisms linking the hyperosmotic microenvironment of the ocular surface with inflammation in DED remain to be further investigated.

In recent years, immunometabolism research has provided new insights into the mechanisms underlying DED. Metabolomic studies of tears from DED patients have demonstrated significant upregulation of glycolysis and gluconeogenesis^17^ and elevated expression of glycolytic enzymes,^18^ including alpha-enolase 1 (ENO1), peroxiredoxin 6 (PRDX6), phosphoglycerate mutase 1 (PGAM1), M-type pyruvate kinase (PKM), and glyceraldehyde-3-phosphate dehydrogenase (GAPDH).^19^ The corneas of DED patients were shown to exhibit a state of high-energy metabolism, with metabolic reprogramming similar to that of the Warburg effect observed in tumors.^20^^,^^21^ This phenomenon closely resembles the metabolic characteristics of immune cells: In their resting state, immune cells primarily rely on oxidative phosphorylation for energy supply; however, under inflammatory stimulation, their metabolism shifts to glycolysis to rapidly generate adenosine triphosphate (ATP) and pro-inflammatory mediators.^22^^,^^23^ Glycolysis provides the energy for inflammation, but its metabolic intermediates, including nicotinamide adenine dinucleotide (NAD+), also directly regulate signaling pathways, including nuclear factor kappa B (NF-κB),^22^ that are closely associated with inflammatory disease mechanisms of systemic lupus erythematosus^24^ and rheumatoid arthritis.^25^ These findings suggest that the hyperosmotic environment of the DED ocular surface may exacerbate local inflammatory responses by modulating glycolytic metabolism.

6-phosphofructo-2-kinase/fructose-2,6-biphosphatase (PFKFB) is a crucial regulator in the glycolytic regulatory network. PFKFB3 is a member of the bifunctional PFKFB enzyme family and has the highest kinase activity, with a kinase/phosphatase activity ratio of 710:1.^26^ PFKFB3 significantly accelerates the glycolytic rate by generating fructose-2,6-bisphosphate (F2,6BP), which activates the rate-limiting enzyme phosphofructokinase 1 (PFK1).^26^ Studies showed that PFKFB3-dependent glycolytic reprogramming amplified inflammatory signals by activating NF-κB phosphorylation, thereby forming a positive feedback loop between metabolism and inflammation.^26^^–^^30^ PFKFB3 serves both as an engine for metabolic reprogramming and as a hub for amplifying inflammatory signals. Notably, hyperosmotic environments, such as high levels of NaCl, were found to specifically enhance PFKFB3 activity.^31^ This led us to propose that glycolysis in the ocular surface microenvironment of DED patients may promote inflammatory responses.

The core pathogenesis of DED is currently focused on tear film hyperosmolarity and ocular surface inflammation, but the potential link between corneal glycolysis and inflammatory processes remains unexplored. By systematically interrogating glycolytic and inflammatory mechanisms, we postulated that aberrant glycolytic activity within the ocular surface microenvironment may exacerbate inflammatory cascades in DED. To experimentally confirm this hypothesis, we identified pivotal regulatory genes in human corneal epithelial cells (HCECs) under hyperosmotic stress and further delineated their functional roles in driving both glycolytic reprogramming and NF-κB signaling pathway activation.

## Methods

### Cell Culture

HCECs were obtained from Sigma-Aldrich (St. Louis, MO, USA) and cultured in Dulbecco's Modified Eagle Medium/Nutrient Mixture F-12 (DMEM/F-12; Thermo Fisher Scientific, Waltham, MA, USA) supplemented with 10% fetal bovine serum (AusgeneX; Molendinar, Queensland, Australia) and 1% Gibco penicillin–streptomycin (Thermo Fisher Scientific). Cells were maintained at 37°C in a humidified incubator with 5% CO_2_.

Hyperosmotic stress in HCECs was induced by supplementing normal culture medium with 69-mM NaCl (Sinopharm Chemical Reagent Co., Ltd., Shanghai, China) for 24 hours, establishing an in vitro DED cellular model with a total osmolality of 450 mOsM.^9^

### Western Blot Analysis

Total protein was isolated from HCECs using radioimmunoprecipitation assay (RIPA) buffer (Solarbio, Beijing, China). The protein concentration was determined by a bicinchoninic acid (BCA) assay. Proteins were separated using 10% SDS-PAGE gels and then transferred onto 0.22-µm nitrocellulose membranes (Pall Corporation, Port Washington, NY, USA). The membranes were blocked with a rapid blocking buffer at room temperature for 15 minutes and then incubated overnight with primary antibodies at 4°C. The following day, the membranes were incubated with secondary antibodies at 37°C for 2 hours. Supplementary Table S1 provides a list of the primary and secondary antibodies used in this study. Protein bands were visualized using an enhanced chemiluminescence kit (Bio-Rad Laboratories, Hercules, CA, USA) on a chemiluminescence imaging system.

### Reverse Transcription Quantitative Polymerase Chain Reaction

Total RNA was isolated using a FastPure Cell/Tissue Total RNA Isolation Kit V2 (Vazyme, Nanjing, China) according to the manufacturer's protocol. First-strand cDNA was synthesized from total RNA using HiScript III All-in-One RT SuperMix for qPCR (Vazyme). Quantitative PCR amplification was performed by using ChamQ Universal SYBR qPCR master mix (Vazyme) and specific primers (listed in Supplementary Table S2). Relative mRNA expression levels were normalized to β-actin using the comparative threshold cycle (ΔΔCt) method, calculated as 2^−ΔΔCt^.

### Cell Viability Assay

Cell viability was measured using a Cell Counting Kit 8 (CCK-8; DOJINDO Laboratories, Kumamoto, Japan) according to the manufacturer's instructions. HCECs were seeded in 96-well plates at 5 × 10^3^ cells per well and cultured for 24 hours. The cells were then treated with 3-(3-pyridinyl)-1-(4-pyridinyl)-2-propen-1-one (3PO) at different concentrations and cultured for another 24 hours. Then, 10 µL of CCK-8 reagent was added to each well, and cells were incubated for an additional 2 hours. The absorbance was measured at 450 nm using a microplate reader.

### Cell Transfection

A *PFKFB3* overexpression plasmid and *PFKFB3*-targeting small interfering RNA (siRNA) were transiently transfected into HCECs using Invitrogen Lipofectamine 3000 (Thermo Fisher Scientific), with the empty vector serving as the negative control. To generate the transfection complexes, 1.5 µg of plasmid DNA or 50-nM siRNA was diluted in 250 µL of Gibco Opti-MEM medium (Thermo Fisher Scientific), and 3.75 µL of Lipofectamine 3000 was separately mixed with 250 µL of Opti-MEM. After incubating at room temperature, the two solutions were combined and incubated for 15 minutes at room temperature. The DNA–lipid complexes were then added to cells cultured in antibiotic-free medium. Transfection efficiency was validated via western blot 48 hours post-transfection.

### Lactate Measurement

Extracellular lactate levels were quantified using a lactic dehydrogenase assay kit (Jiancheng Bioengineering Institute, Nanjing, China) in strict accordance with the manufacturer's protocol. Blank, standard, and test samples were prepared using 0.02 mL of double-distilled water (ddH_2_O), 0.02 mL of 3 mmol/L lactate standard, and 0.02 mL of the test sample, respectively. Then, 1.0 mL of the enzyme working solution and 0.2 mL of a chromogenic agent were added to each tube. After thorough mixing, the reactions were incubated at 37°C for 10 minutes and then terminated with 2 mL of a termination solution. The absorbance values (*A*) were measured at 530 nm using a 1-cm pathlength cuvette with ddH_2_O as a zero reference. The formula for determining the lactic acid concentration in the liquid samples was as follows:

$$\begin{array}{ccc} \mathrm{Lactate}\; & = & \;\left(A\;\mathrm{sample}-A\;\mathrm{blank}\right)/\left(A\;\mathrm{standard}-A\;\mathrm{blank}\right)\\ & & \times \;\mathrm{Standard}\;\;\mathrm{concentration}\;\left(3\;\mathrm{mmol}/L\right) \end{array}$$

### Statistical Analysis

Data are presented as mean ± standard deviation (SD) and derived from at least three independent biological replicates, with triplicate technical measurements performed per biological replicate. Comparisons between two groups were analyzed using Student's *t*-test. One-way analysis of variance (ANOVA) followed by Tukey's multiple-comparison test was used to compare more than two groups. Statistical analyses were conducted using Prism 8 (GraphPad, Boston, MA, USA). Next-generation RNA sequencing (RNA-seq) data processing and Kyoto Encyclopedia of Genes and Genomes (KEGG) pathway enrichment analyses were performed by Guangke Ande Biotechnology Co., Ltd. (Hangzhou, China). Statistical significance was denoted as follows: *P < 0.05, **P < 0.01, ***P < 0.001, and ****P < 0.0001.

## Results

### Hyperosmotic Stress Activates Glycolysis and Inflammation in HCECs

**Figure 1.**
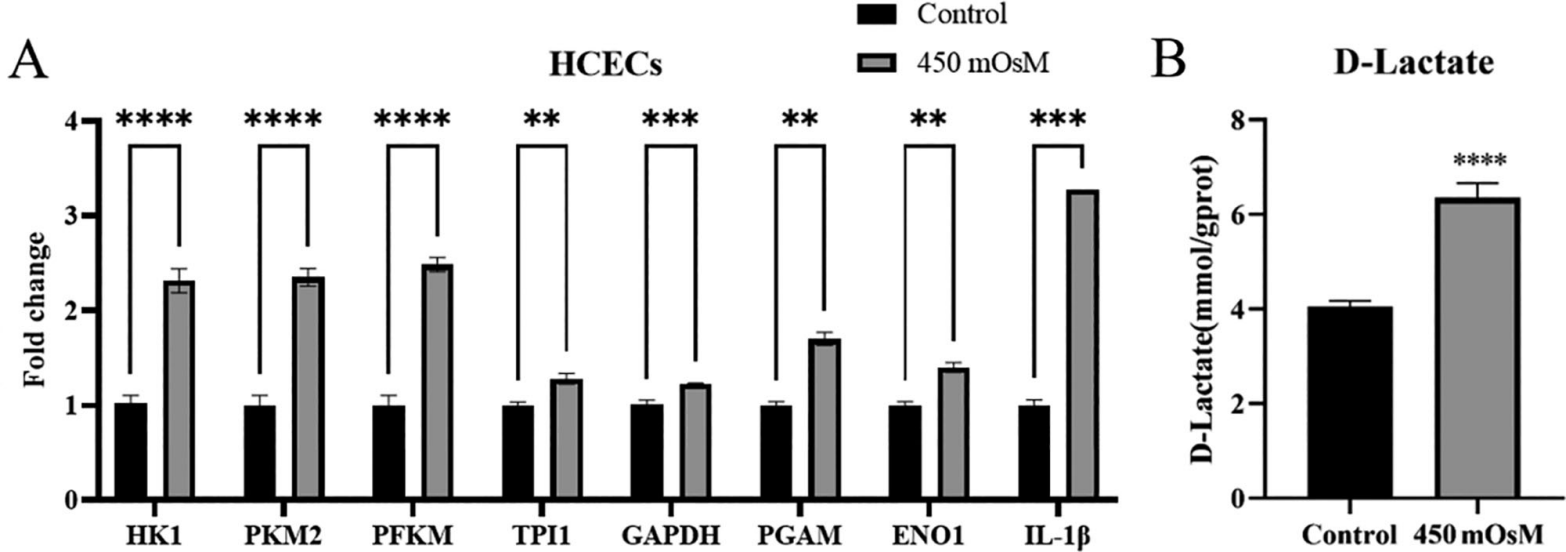
Hyperosmotic stress promotes upregulation of glycolysis-associated factors and inflammatory-associated factor expression in HCECs. (**A**) The mRNA expression of the glycolytic enzymes *HK1*, *PKM2*, *PFKM*, *TPI1*, *GAPDH*, *PGAM*, and *ENO1* and the pro-inflammatory cytokine IL-1β were quantified using RT-qPCR (*n* = 3). (**B**) Extracellular lactate secretion was measured with a lactate assay kit (*n* = 3). The data are presented as mean ± SD. Statistical significance was assessed using a two-tailed unpaired *t*-test with the following thresholds: *P < 0.05, **P < 0.01, ***P < 0.001, and ****P < 0.0001.

To explore whether hyperosmolarity activates glycolytic and inflammatory pathways in HCECs, we generated a DED cellular model by culturing HCECs in culture medium supplemented with 69-mM NaCl for 24 hours (450 mOsM).^9^ RT-qPCR analysis (Fig. 1A) revealed that, compared to the isotonic control, there was significant transcriptional upregulation in the hyperosmotic-induced HCECs of the following glycolytic enzymes (312 mOsM): hexokinase 1 (*HK1*), pyruvate kinase M2 (*PKM2*), phosphofructokinase muscle-type (*PFKM*), triosephosphate isomerase 1 (*TPI1*), *GAPDH*, *PGAM*, and *ENO1*. Lactate levels were quantified and showed a 1.5-fold increase in extracellular lactate secretion under hyperosmotic conditions (Fig. 1B). At the same time, hyperosmotic stress induced a 3.5-fold upregulation of *IL-1β* mRNA expression (Fig. 1A), confirming the activation of inflammatory pathways.

### Transcriptomic Profiling Reveals Upregulation of the Glycolytic Gene *PFKFB3* and NF-κB–Related Genes

**Figure 2.**
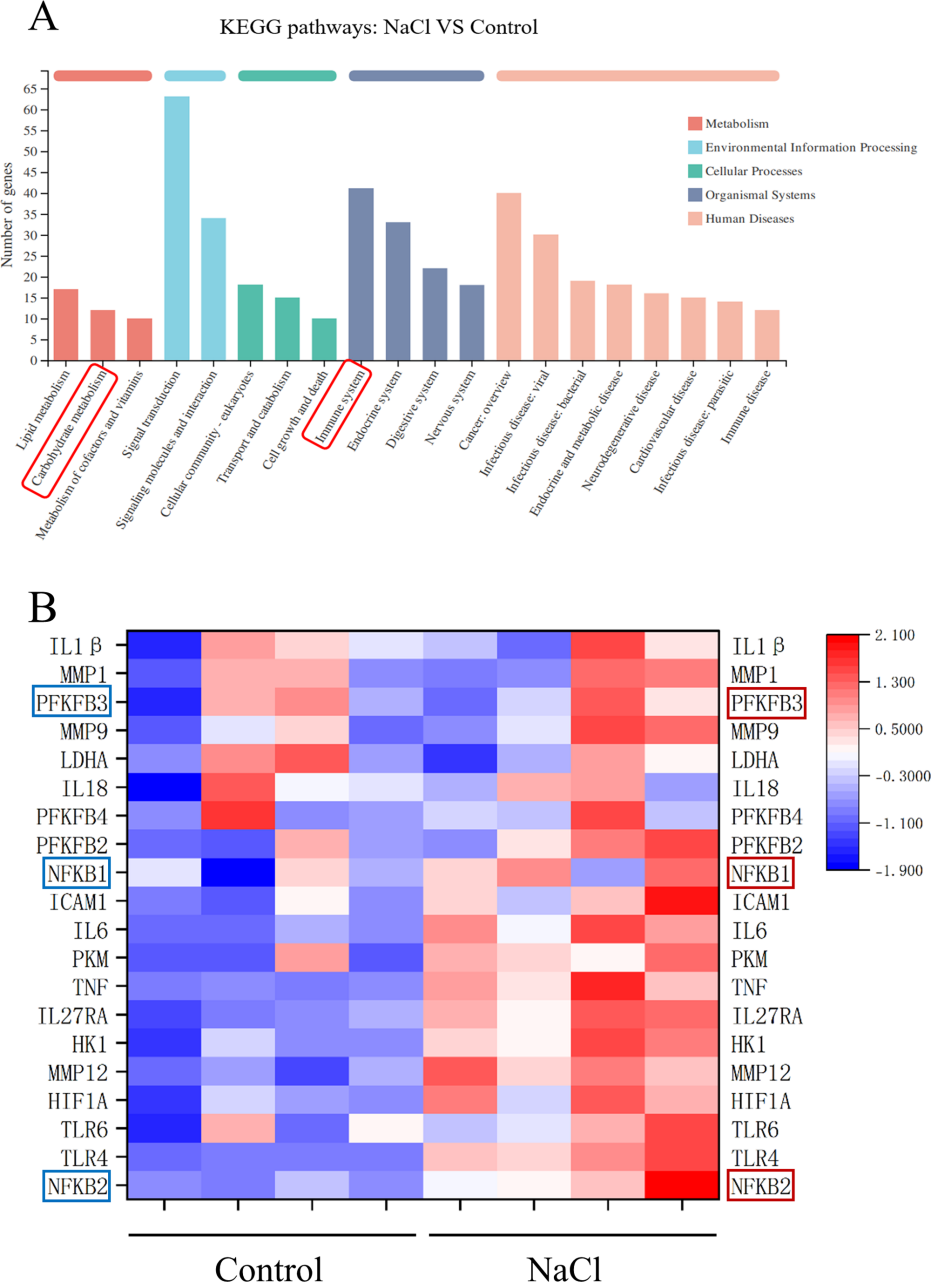
RNA-seq and KEGG pathway enrichment analysis results of hyperosmotic HCECs. (**A**) A KEGG pathway enrichment analysis was conducted to compare the hyperosmotic group with the control group, with a particular focus on the pathways related to carbohydrate metabolism and the immune system. (**B**) The heatmap illustrates the differential expression of genes associated with carbohydrate metabolism and the immune system in hyperosomotic-induced HCECs compared to control HCECs.

We conducted next-generation RNA-seq and KEGG pathway enrichment analysis on HCECs cultured under isotonic and hyperosmotic conditions. The KEGG pathway analysis (Fig. 2A) revealed significant enrichment of pathways related to carbohydrate metabolism and the immune system in the hyperosmotic group compared to the control group. A further examination of the metabolic and immune signatures indicated a marked upregulation of the glycolytic regulator *PFKFB3* and NF-κB–related genes in hyperosmotic-induced HCECs (Fig. 2B). Building on prior evidence that PFKFB3-driven glycolytic reprogramming promoted endothelial inflammation via NF-κB signaling,^29^ we hypothesized that hyperosmotic-induced overexpression of *PFKFB3* may mechanistically regulate inflammation associated with DED through the NF-κB pathway.

### Activation of the Glycolytic Enzyme PFKFB3 and NF-κB Inflammatory Pathway in a Hyperosmotic DED Model

**Figure 3.**
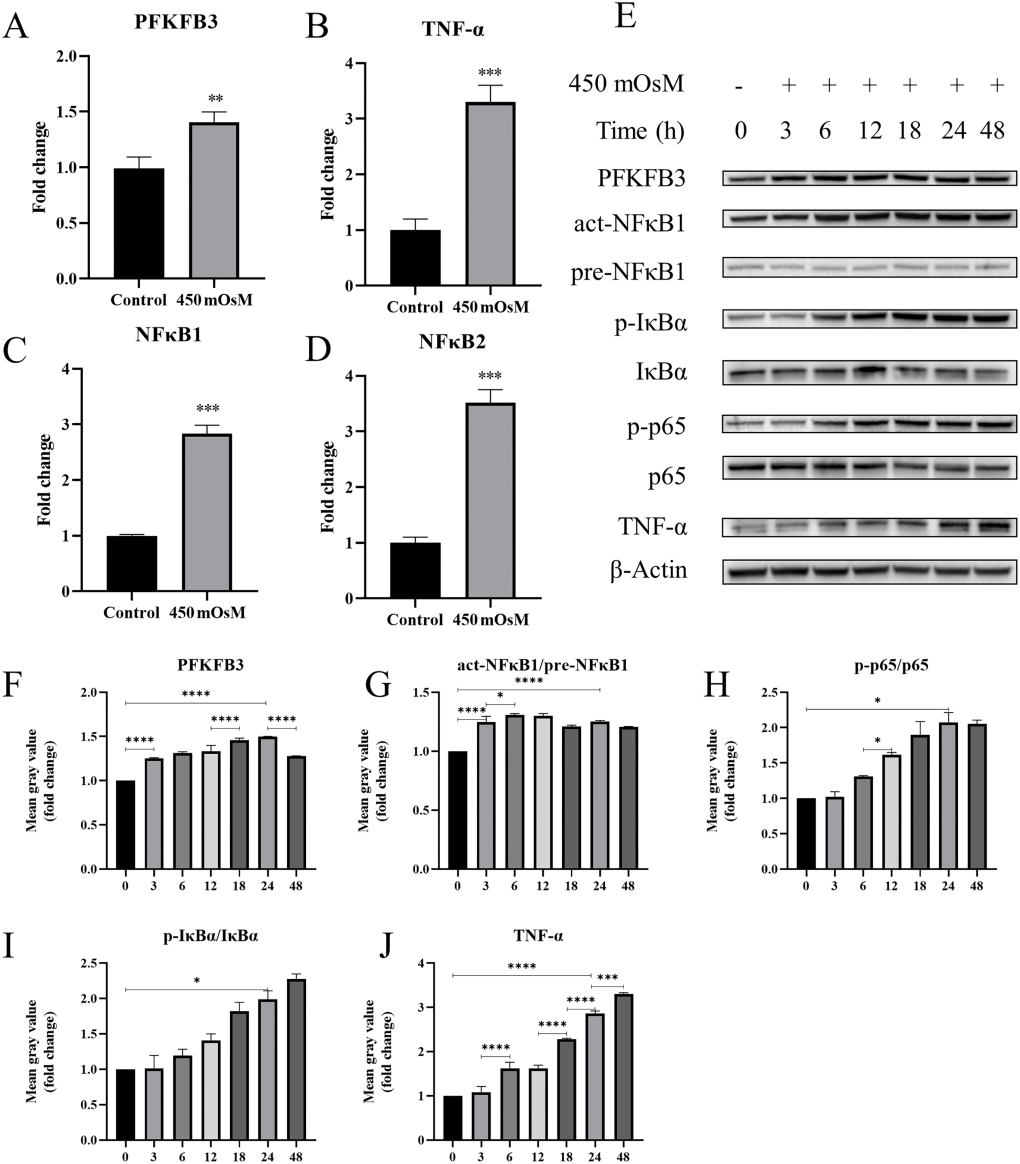
Hyperosmotic stress upregulates PFKFB3 and NF-κB signaling in HCECs. (**A**–**D**) The mRNA expression of the glycolytic enzyme PFKFB3 (**A**) and NF-κB pathway–related genes, *TNF-α* (**B**), *NFκB1* (**C**), and *NFκB2* (**D**) in HCECs exposed to hyperosmotic conditions was quantified using RT-qPCR (*n* = 3). (**E**) Western blot analysis was conducted to assess the expression of PFKFB3, act-NFκB1, pre-NFκB1, p-IκBα, IκBα, p-p65, p65, TNF-α, and β-actin in HCECs under hyperosmotic conditions (450-mOsM NaCl) during a time course at 0, 3, 6, 12, 18, 24, and 48 hours (*n* = 3). (**F**–**J**) A normalized grayscale intensity analysis of the protein bands in **E** was performed. Data are presented as mean ± SD. Statistical significance was determined using one-way ANOVA with Tukey's multiple comparisons test, and significance levels are indicated as follows: *P < 0.05, **P < 0.01, ***P < 0.001, and ****P < 0.0001.

RT-qPCR analysis validated our hypothesis that hyperosmotic stimulation significantly upregulated mRNA expression levels in HCECs of *PFKFB3* (Fig. 3A), *TNF-α* (Fig. 3B), *NFκB1* (Fig. 3C), and *NFκB2* (Fig. 3D). This supported the transcriptional activation of both *PFKFB3* and NF-κB signaling pathway-related genes. Furthermore, western blotting revealed time-dependent changes in protein expression in hyperosmotic-induced HCECs (Figs. 3E–3I). PFKFB3 protein levels progressively increased with hyperosmotic exposure, peaking at approximately 24 hours post-treatment (Fig. 3F). We then performed a western blot analysis to examine the protein levels of activated NF-κB1 (act-NFκB1), precursor NF-κB1 (pre-NFκB1), phosphorylated IκBα (p-IκBα), IκBα, phosphorylated p65 (p-p65), p65, TNF-α, and β-actin in HCECs treated with 450-mOsM hypertonic NaCl (Fig. 3E). We observed that PFKFB3 levels (Fig. 3F), the ratio of act-NFκB1 to pre-NFκB1 (Fig. 3G), the ratio of p65 to total p65 (Fig. 3H), and the ratio of phosphorylated p-IκBα to IκBα (Fig. 3I) all gradually increased over the treatment time course. Additionally, TNF-α protein levels exhibited sustained upregulation over time (Fig. 3J). Collectively, these findings established that hyperosmotic stress induced a coordinated, time-dependent activation of PFKFB3-mediated glycolytic reprogramming and NF-κB inflammatory signaling in HCECs.

### *PFKFB3* Overexpression Augments NF-κB Pathway Activation in a Hyperosmotic DED Model

**Figure 4.**
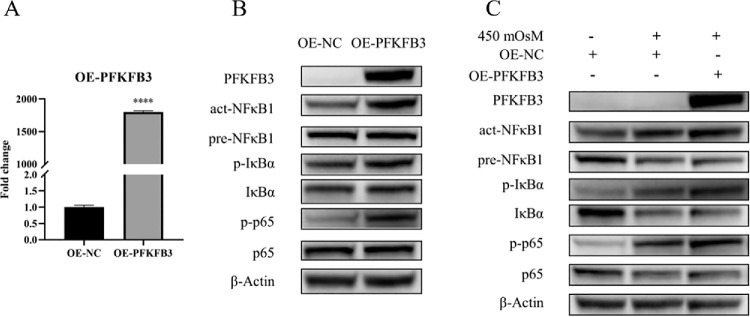
NF-κB activation induced by *PFKFB3* overexpression in a DED model. (**A**) RT-qPCR was conducted to assess *PFKFB3* mRNA levels in HCECs transfected with an *PFKFB3* overexpression vector compared to the empty vector control (*n* = 3). (**B**) Western blot analysis was performed to evaluate the levels of PFKFB3, act-NFκB1, pre-NFκB1, p-IκBα, IκBα, p-p65, p65, and β-actin in both the *PFKFB3* overexpression and empty vector control groups, with the results representative of three biological replicates (*n* = 3). (**C**) Western blot analysis was conducted to examine the expression of *PFKFB3* and components of the NF-κB pathway to compare the PFKFB3 overexpression group with the empty vector control group in the DED model (*n* = 3). Data are presented as mean ± SD, and statistical significance was determined using independent samples *t*-tests, with ****P < 0.0001 indicating high significance. NC, normal control.

To investigate the regulation of NF-κB signaling by PFKFB3, we constructed a *PFKFB3* overexpression plasmid (OE-PFKFB3) and transfected HCECs for 48 hours. RT-qPCR analysis indicated an approximate 1800-fold increase in *PFKFB3* mRNA levels in OE-PFKFB3–transfected cells compared to the empty vector control (Fig. 4A). Furthermore, western blot analysis confirmed significant upregulation of PFKFB3 protein expression in OE-PFKFB3–transfected HCECs (Fig. 4B). Notably, *PFKFB3* overexpression enhanced the protein levels of NF-κB pathway activation markers, including act-NFκB1, p-IκBα, and p-p65 (Fig. 4B). Consistent with these findings, in the 450-mOsM NaCl-induced hyperosmotic DED model, OE-PFKFB3 transfection further amplified PFKFB3 protein expression and potentiated NF-κB pathway activation (Fig. 4C). This functional interplay suggests a causal role for PFKFB3-driven glycolytic reprogramming in hyperosmotic-induced inflammatory signaling.

### Pharmacological Inhibition of PFKFB3 Attenuates Glycolytic Reprogramming and NF-κB Pathway Activation in a Hyperosmotic DED Model

**Figure 5.**
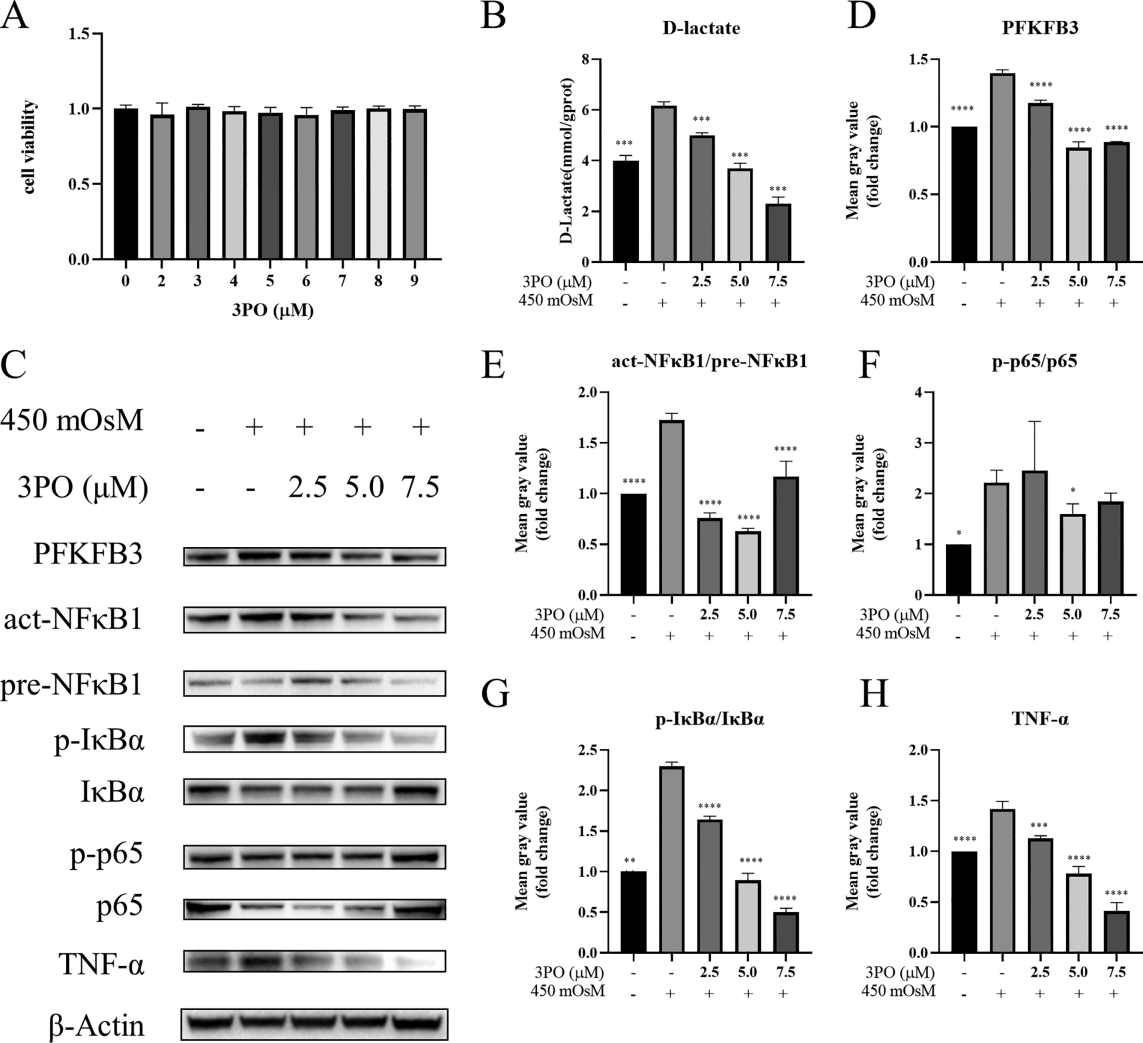
3PO-mediated PFKFB3 inhibition suppresses lactate synthesis and NF-κB signaling in a DED model. (**A**) Cell viability of HCECs treated with 0 to 9 µM 3PO for 24 hours was assessed using a CCK-8 assay (*n* = 3). (**B**) Lactate secretion in HCECs treated with 2.5, 5.0, or 7.5 µM 3PO under hyperosmotic conditions (450-mOsM NaCl) was quantified using a lactate assay (*n* = 3). (**C**) Western blot analysis was performed to evaluate the levels of PFKFB3, act-NFκB1, pre-NFκB1, p-IκBα, total IκBα, p-p65, total p65, TNF-α, and β-actin proteins in HCECs treated with different concentrations of 3PO (*n* = 3). (**D**–**H**) A normalized grayscale intensity analysis of western blot protein bands was conducted. Data are expressed as mean ± SD. Statistical significance was determined using one-way ANOVA with Tukey's multiple comparisons test, and significance levels are indicated as follows: *P < 0.05, **P < 0.01, ***P < 0.001, and ****P < 0.0001.

To investigate whether PFKFB3 promoted NF-κB activation through glycolytic upregulation, hyperosmotic-induced HCECs were treated with 3PO, a specific inhibitor of PFKFB3. An initial cytotoxicity assessment revealed no significant changes in cell viability after 24 hours across a concentration range of 0 to 9 µM 3PO compared to the control group (Fig. 5A). Consequently, concentrations of 2.5, 5, and 7.5 µM 3PO were selected for subsequent experiments. Treatment with 3PO resulted in a concentration-dependent reduction in lactate secretion compared to untreated hyperosmotic controls (Fig. 5B), thus confirming that the PFKFB3-mediated glycolytic flux was inhibited by 3PO. Western blot analysis (Fig. 5C) indicated dose-dependent decreases in PFKFB3 protein levels (Fig. 5D), phosphorylation of IκBα (Fig. 5G), and TNF-α protein levels (Fig. 5H) in the 3PO-treated groups. The greatest suppression of NF-κB1 activation (Fig. 5E) and p65 phosphorylation (Fig. 5F) was observed at a concentration of 5 µM 3PO. Collectively, these findings demonstrated that pharmacological inhibition of PFKFB3 with 3PO effectively disrupted hyperosmotic-induced glycolytic reprogramming and subsequent activation of the NF-κB pathway, thereby alleviating the inflammatory response in the DED model.

### Genetic Knockdown of *PFKFB3* Suppresses NF-κB Pathway Activation in a Hyperosmotic DED Model

**Figure 6.**
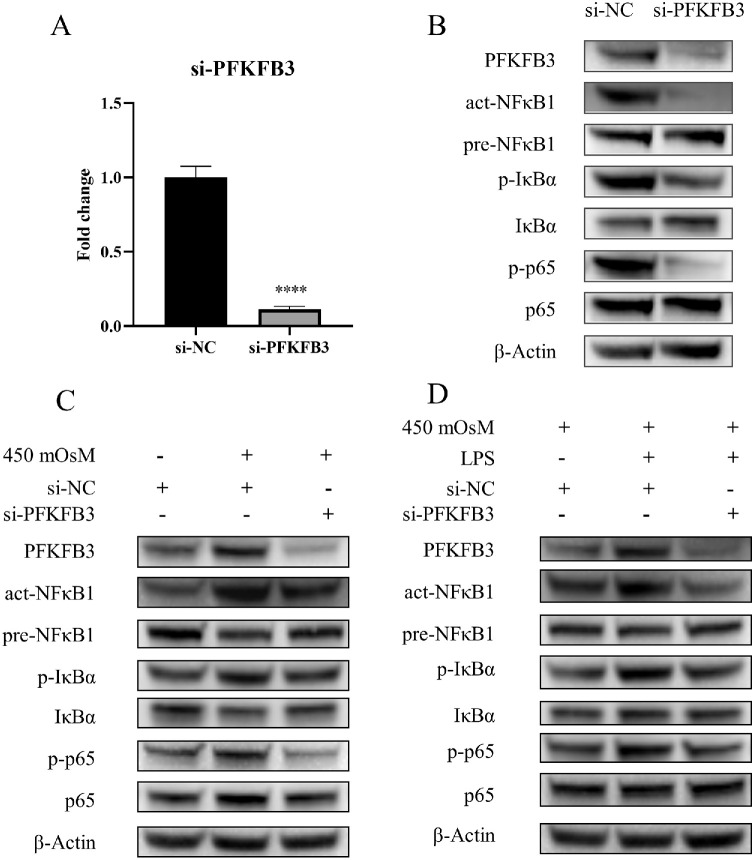
Targeting PFKFB3 by genetic knockdown mitigates NF-κB pathway hyperactivation in a DED model. (**A**) The expression of *PFKFB3* mRNA in HCECs transfected with si-*PFKFB3* or scrambled si-NC for 48 hours was quantified using RT-qPCR (*n* = 3). (**B**) Western blot analysis was performed to assess the expression of PFKFB3, act-NFκB1, pre-NFκB1, p-IκBα, total IκBα, p-p65, total p65, and β-actin in both the si-NC and si-*PFKFB3* groups (*n* = 3). (**C**) The western blot results validated the effects of *PFKFB3* knockdown on NF-κB pathway activation in the 450-mOsM NaCl–induced DED model (*n* = 3). (**D**) Western blot analysis confirmed that *PFKFB3* knockdown attenuated LPS-induced activation of the NF-κB signaling pathway (*n* = 3). Data are presented as mean ± SD, and statistical significance was determined using the independent samples *t* test, with ****P < 0.0001 indicating high significance.

To genetically validate the PFKFB3-mediated regulation of NF-κB signaling, HCECs were transfected with *PFKFB3*-targeting siRNA. RT-qPCR analysis confirmed the efficient knockdown of *PFKFB3*, with only 11.3% residual mRNA expression compared to the non-targeting scramble siRNA control (si-NC) 48 hours post-transfection (Fig. 6A). Western blot analysis demonstrated that selective knockdown of *PFKFB3* significantly reduced protein levels of PFKFB3 and concomitantly diminished the activation of NF-κB pathway components, including act-NFκB1, p-IκBα, and p-p65 (Fig. 6B). In the hyperosmotic DED model, silencing *PFKFB3* attenuated NaCl-induced activation of NF-κB (Fig. 6C). To further corroborate this regulatory relationship, HCECs were treated with 10 µg/mL lipopolysaccharide (LPS), a canonical NF-κB agonist. LPS treatment upregulated both PFKFB3 levels and NF-κB activation. However, *PFKFB3* knockdown effectively counteracted the LPS-driven stimulation of the NF-κB pathway (Fig. 6D). These genetic manipulations conclusively demonstrated that depleting *PFKFB3* inhibited NF-κB signaling across multiple inflammatory stimuli.

## Discussion

DED is a multifactorial chronic inflammatory disorder of the ocular surface that significantly impairs patient quality of life. Although inflammatory mechanisms are critical in the pathogenesis of DED, a comprehensive understanding of these mechanisms has remained elusive, highlighting the need for further investigations that can inform novel therapeutic strategies. Corneal epithelial cells exhibit high glycolytic metabolic activity that dynamically adapts to fluctuating energy demands, which is crucial for maintaining ocular surface homeostasis.^32^ Emerging evidence has highlighted the intricate interplay between glycolytic metabolic reprogramming and inflammatory processes.^33^ Clinical studies found a marked increase in ocular surface glycolysis in DED patients compared to healthy individuals.^18^^,^^19^^,^^34^ However, the causal relationship and mechanistic links between glycolytic remodeling and ocular surface inflammation in DED are still poorly characterized; thus, a systematic investigation is needed.

In this study, we first established a hyperosmotic-induced DED cellular model, which exhibited significantly upregulated mRNA expression of key glycolytic enzymes and the inflammatory cytokine IL-1β and was accompanied by elevated lactate production. These findings suggested a synergistic activation between glycolysis and DED-associated inflammation. Transcriptomic sequencing and KEGG pathway enrichment analysis further revealed that hyperosmotic conditions specifically upregulated *PFKFB3* and NF-κB pathway-related genes. Notably, prior studies identified NF-κB binding sites within the *PFKFB3* promoter^35^ and showed that the NF-κB pathway—a canonical regulator of inflammation—participated in DED pathogenesis by modulating the synthesis and release of cytokines, chemokines, and adhesion molecules.^36^^–^^38^ Building on evidence that hyperosmotic environments activate *PFKFB3* transcription in cancer cells,^39^ we hypothesized that hyperosmotic stress in DED may drive NF-κB–mediated inflammatory cascades via PFKFB3-dependent glycolytic reprogramming.

The experimental results demonstrated that hyperosmotic stress promoted glycolytic reprogramming through time-dependent *PFKFB3* upregulation, consistent with its osmotic stress response mechanisms in other cell types.^31^ Crucially, inhibiting PFKFB3 levels, either pharmacologically with 3PO or through siRNA-mediated knockdown, effectively suppressed the glycolytic flux, inhibited NF-κB nuclear translocation, and reduced downstream cytokine production. Conversely, *PFKFB3* overexpression markedly enhanced NF-κB activity. These results confirmed PFKFB3 as a critical node linking glycolysis and inflammatory signaling, and identified for the first time a functional PFKFB3–NF-κB axis in DED.

Mechanistically, *PFKFB3* inhibition may alleviate hyperosmotic stress-driven inflammatory responses through the metabolic regulation of the NF-κB pathway and attenuate hyperosmotic-induced inflammation by reducing lactate accumulation. Végran et al.^40^ reported that lactate, a glycolytic byproduct, stimulated NF-κB signaling in endothelial cells via monocarboxylate transporters, triggering IκBα phosphorylation and degradation with subsequent NF-κB/IL-8 pathway activation.^40^ Our data corroborated that PFKFB3 inhibition suppressed lactate levels, suggesting that lactate may modulate PFKFB3-dependent NF-κB activation.^41^

The upstream regulatory mechanisms of PFKFB3 still warrant further investigation. First, the p38/MK2 pathway has been implicated in NaCl-induced hyperosmotic activation of the *PFKFB3* promoter.^31^ MAPK-activated protein kinase 2, a key component of the MAPK pathway, was found to promote *PFKFB3* gene transcription and allosteric activation through phosphorylation of specific substrate residues, enhancing glycolytic flux. Second, hypoxia-inducible factor 1 alpha (HIF-1α) may act as a critical transcriptional regulator of *PFKFB3*. Hypoxia is the primary activator of HIF-1α; however, emerging evidence has indicated that non-hypoxic stimuli, including cytokines (e.g., TNF-α, IL-1β) and inflammatory mediators (e.g., nitric oxide), also upregulate HIF-1α under normoxic conditions.^42^^–^^44^ Third, PFKFB3 is a target of adenosine monophosphate (AMP)-activated protein kinase (AMPK), a cellular energy sensor. AMPK activation was shown to rapidly increase glucose uptake and glycolysis, with PFKFB3 phosphorylation and activation proposed as determinants of AMPK-driven glycolytic activity.^45^ Thus, elucidating the precise regulatory mechanisms of PFKFB3 and its downstream effects on the NF-κB pathway in DED remains a priority for future research.

Although this study established the PFKFB3–NF-κB axis as pivotal in DED inflammation, several unresolved questions remain. For example, the upstream regulators of PFKFB3 in ocular surface cells require detailed characterization. Furthermore, systemic PFKFB3 inhibitors may cause severe off-target toxicities in multiple organ systems, significantly hindering their clinical development. Topical ocular therapy offers a promising means of mitigating these systemic toxicity risks. Localized delivery mechanisms, including topical formulations (eye drops, gels, ointments), ocular local injections, and sustained-release implant systems, dramatically minimize systemic drug exposure and thereby may effectively circumvent the associated off-target effects. By restricting pharmacologically active drugs to intraocular target tissues, the therapeutic index and safety profile can be enhanced while maintaining therapeutic efficacy. Translating this therapeutic concept into viable clinical therapies will require rigorous clinical evaluation of PFKFB3 inhibitors with careful management of any residual off-target effects.

In summary, our study demonstrated that both PFKFB3 and the NF-κB signaling pathway are upregulated in hyperosmolarity-induced HCECs, with *PFKFB3* overexpression directly promoting NF-κB pathway activation. By pharmacologically inhibiting PFKFB3 with 3PO, hyperosmotic stress-induced glycolytic activation and NF-κB signaling were effectively suppressed and inflammatory responses were ameliorated. These findings were further corroborated by genetic suppression of *PFKFB3*, which achieved comparable NF-κB pathway inhibition. These results demonstrated that PFKFB3 regulates dry eye inflammation by activating the NF-κB pathway through glycolytic metabolic reprogramming, highlighting PFKFB3 as a potential therapeutic target for DED treatment.

## Supplementary Material

Supplement 1
