# Supplementary material for: PFKFB3-Mediated Glycolytic Metabolic Reprogramming Regulates Inflammatory Response in Dry Eye Disease
Source: Invest Ophthalmol Vis Sci. 2025 Aug 29;66(11):76. doi: 10.1167/iovs.66.11.76 (PMC12400978; doi:10.1167/iovs.66.11.76)
Supplement: Supplement 1 [file iovs-66-11-76_s001.pdf]

Supplementary Material

Supplementary Table S1. [Experimental Antibody Information]

Supplementary Table S2. [Primer Sequence for Experiments]

Supplementary Table S1 Experimental Antibody Information

Antibody	Manufacturer	Catalog Number
anti-β-Actin	HuaAn biotechnology	10001
anti-PFKFB3	Abcam	Ab181861
anti-IκBα	Cell Signaling Technology	4814
anti-phospho-IκBα	Cell Signaling Technology	2859
anti-NFκB p65	Cell Signaling Technology	8242
anti- phospho-NFκB p65	Cell Signaling Technology	3033
anti-TNF-α	Cell Signaling Technology	3707
Rabbit anti Goat IgG-HRP	Proteintech	SA00001-4
Goat anti Mouse IgG-HRP	HuaAn biotechnology	1006
Goat anti Rabbit IgG-HRP	HuaAn biotechnology	1001

Supplementary Table S2 Primer Sequence for Experiments

Name	Sequence
Human-NFκB1-Forward Primer	5'-GGTGCGGCTCATGTTTACAG-3'
Human-NFκB1- Reverse Primer	5'-GATGGCGTCTGATACCACGG-3'
Human-NFκB2-Forward Primer	5'-CCATGACAGCAAATCTCC-3'
Human-NFκB2- Reverse Primer	5'-TAAACTTCATCTCCACCCC-3'
Human-PFKFB3-Forward Primer	5'-TTGGCGTCCCCACAAAAGT-3'
Human-PFKFB3- Reverse Primer	5'-AGTTGTAGGAGCTGTACTGCTT-3'
Human-TNF-α-Forward Primer	5'-GAGGCCAAGCCCTGGTATG-3'
Human- TNF-α- Reverse Primer	5'-CGGGCCGATTGATCTCAGC-3'
Human-si-PFKFB3-Forward Primer	5'-GAAGAGGAUCAGUUGCUAU-3'
Human-si-PFKFB3- Reverse Primer	5'-AUAGCAACUGAUCCUCUUC-3'
Human-ACTIN-Forward Primer	5'-CACCATTGGCAATGAGCGGTTC-3'
Human-ACTIN- Reverse Primer	5'-AGGTCTTTGCGGATGTCCACGT-3'
